# EMR-integrated minimal core dataset for routine health care and multiple research settings: A case study for neuroinflammatory demyelinating diseases

**DOI:** 10.1371/journal.pone.0223886

**Published:** 2019-10-15

**Authors:** Sophia von Martial, Tobias J. Brix, Luisa Klotz, Philipp Neuhaus, Klaus Berger, Clemens Warnke, Sven G. Meuth, Heinz Wiendl, Martin Dugas

**Affiliations:** 1 Institute of Medical Informatics, University of Münster, Münster, Germany; 2 Department of Neurology, University of Münster, Münster, Germany; 3 Institute of Epidemiology and Social Medicine, University of Münster, Münster, Germany; 4 Department of Neurology, University of Köln, Köln, Germany; Heinrich-Heine-Universitat Dusseldorf, GERMANY

## Abstract

Although routine health care and clinical trials usually require the documentation of similar information, data collection is performed independently from each other, resulting in redundant documentation efforts. Standardizing routine documentation can enable secondary use for medical research. Neuroinflammatory demyelinating diseases (NIDs) represent a heterogeneous group of diseases requiring further research to improve patient management. The aim of this work is to develop, implement and evaluate a minimal core dataset in routine health care with a focus on secondary use as case study for NIDs. Therefore, a draft minimal core dataset for NIDs was created by analyzing routine, clinical trial, registry, biobank documentation and existing data standards for NIDs. Data elements (DEs) were converted into the standard format Operational Data Model, semantically annotated and analyzed via frequency analysis. The analysis produced 1958 DEs based on 864 distinct medical concepts. After review and finalization by an interdisciplinary team of neurologists, epidemiologists and medical computer scientists, the minimal core dataset (NID CDEs) consists of 46 common DEs capturing disease-specific information for reuse in the discharge letter and other research settings. It covers the areas of diagnosis, laboratory results, disease progress, expanded disability status scale, therapy and magnetic resonance imaging findings. NID CDEs was implemented in two German university hospitals and a usability study in clinical routine was conducted (participants n = 16) showing a good usability (Mean SUS = 75). From May 2017 to February 2018, 755 patients were documented with the NID CDEs, which indicates the feasibility of developing a minimal core dataset for structured documentation based on previously used documentation standards and integrating the dataset into clinical routine. By sharing, translating and reusing the minimal dataset, a transnational harmonized documentation of patients with NIDs might be realized, supporting interoperability in medical research.

## Introduction

Documentation in routine health care is very heterogeneous and unstructured [1]. Given a certain disease, the captured documentation of two different hospitals will usually differ significantly [2]. But not only in clinical routine care, also across clinical trials or pragmatic trials [3] a low degree of standardization in data collection limits the validity of possible clinically relevant results [4]. This varying documentation hampers the potential of secondary use, which can reduce redundant documentation efforts, resulting in an overall cost reduction [5]. However, a trade-off must be found between extensive data collection as practiced in trials, and the capacity of physicians to document all elements during routine care on top of their daily documentation load.

This problem has been addressed by multiple institutions, including the National Institute of Neurological Disorders and Stroke (NINDS), the National Institute of Health (NIH) and the Clinical Data Interchange Standards Consortium (CDISC) [6–8]. By developing so-called common data elements (CDEs) for various disease entities, such as spinal cord injuries and epilepsy, or the Clinical Data Acquisition Standards Harmonization (CDASH), basic standards for the collection of clinical trial data have been published [9–11].

The NIH defines a CDE as “data element that is common to multiple datasets across different studies” [12]. They represent consented catalogs of metadata, consisting of attributes, permissible values, and response options of a data element [13]. CDEs already are used in clinical trials to enhance data integration from various sources.

However, data integration from electronic medical records (EMRs) is still at an early stage. Data extraction for secondary use or identification of eligible patients still pose a great challenge. Natural language processing on free texts in discharge letters, relying on coding procedures, such as ICD-10-GM coding for billing purposes, as well as semantic annotation are frequently used for the purpose of data extraction [14, 15]. Nevertheless, negations, misspelling, and the purpose of coding hamper the quality of such approaches [16].

The use of CDEs could contribute to solving this problem. However, CDEs and secondary use of data have not yet been established as a fundamental part of documentation processes in clinical routine. For a single-source strategy, integrating data from clinical routine and medical research, proper data quality (structured, harmonized) is obligatory [17]. Improving data collection in clinical routine could save time and decrease financial costs, not only in routine care but also for clinical research [18].

A similar approach to achieve personalized treatment plans in the management of multiple sclerosis (MS) is proposed by Peeters in 2017 [19]. Based on the findable, accessible, interoperable and reusable (FAIR) data principles, Peeters outlines a 4C plan (collect, connect, complete, construct) to collect FAIR data for MS patients in various settings, i.e. in routine and research [19, 20]. Focusing on the minimal requirements for common datasets with regard to pooling and connecting datasets across different institutions, a minimal core dataset could help in this matter [19].

Since minimal core datasets are always related to a single disease or group of diseases, we are focusing our work on the group of neuroinflammatory demyelinating diseases (NIDs). NIDs, such as multiple sclerosis (MS), neuromyelitis optica spectrum disorders (NMOSD) and acute disseminated encephalomyelitis (ADEM) represent a group of diseases that share immune-mediated inflammatory cascades, leading to demyelination in the central nervous system. Heterogeneity regarding pathogenesis, epidemiology, clinical course and treatment options pose a major problem in disease management [21–23]. With respect to disease severity, physical and psychological burden, current research focuses on developing new therapeutic options, performing long-term monitoring of the use of approved and off-label disease modifying therapies (DMTs), overall aiming at improving quality of life for patients suffering from NIDs [24, 25].

This paper presents the development, implementation and evaluation of a minimal core dataset in a clinical setting with a focus on secondary use in a case study for NIDs. Therefore, the definition of a CDE is broadened to “a data element that is common to multiple datasets across different data collection settings”. Based upon a variety of NID documentation and previously published data standards, we propose common data elements for neuroinflammatory demyelinating diseases (NID CDEs), serving as a harmonized and structured data standard in clinical routine. The dataset is implemented as form in the EMRs of two German university hospitals and, to assess usability in clinical routine, a usability evaluation is performed by neurologists. To facilitate documentation processes, several features and the automated generation of a text module for data reuse for discharge letters (DCs) is integrated. Evaluations of the documentation completeness concerning the amount of patients documented with the minimal core dataset and portability to an international registry are performed.

## Materials and methods

### Development of a minimal core dataset

The development of the minimal core dataset initially required researching adequate documentation sources. Knowledge of desired data, as well as its setting and purpose is crucial for the development of CDEs and minimal core datasets. A wide range of different sources is beneficial to promote a future secondary use. Thus, the CDEs can at least be applied in the context of all sources being involved in the development process.

In the NID case study, our clinical and epidemiological cooperation partners provided forms from their research field. In total, material of five different settings was collected covering routine, biobank sampling, data collection for registries, domain-specific CDEs and case report forms (CRFs) from clinical trials:

- Ten anonymized DCs of patients with MS, one anonymized DC of a patient with neuromyelitis optica (NMO) and one of a patient with ADEM, provided by the Department of Neurology, University Hospital Münster, Germany
- Forms of REGIMS, a long-term immunotherapy register (DRKS00007190), initiated by the German Competence Network for Multiple Sclerosis (KKNMS), coordinated by the Institute of Epidemiology and Social Medicine, University Hospital Münster, Germany [26]
- Database fields of the neuroinflammatory biobank of the Department of Neurology, University Hospital Münster, Germany [27]
- Diagnosis and disease characteristics for multiple sclerosis common data elements (MS CDEs) from the NINDS [6]
- CRFs of two clinical trials in MS, investigating the effect of Alemtuzumab (ALAIN, NCT02419378) and Dimethyl fumarate (DIMAT, NCT02461069)

The material was converted into the Operational Data Model (ODM) format with a web-based editor, called ODMedit [28]. ODM is a standard format for exchanging clinical trial data, metadata and administrative data developed by CDISC. ODM supports the arrangement of questions (Items) in groups (ItemGroups) and the definition of answer sets (CodeLists) consisting of single answer options (CodeListItems) [7]. A medical expert identified medical concepts and semantically annotated these concepts with Unified Medical Language System (UMLS) codes [29]. Storing metadata in this standard format and supporting semantic annotation, i.e. mapping of codes of a terminology to medical concepts, may facilitate data integration from various sources [30, 31].

To promote transparency and standardization in medical documentation and support interoperability of EMRs, the Institute of Medical Informatics of the University of Münster established the portal of medical data models (MDMs) [30]. All annotated ODM files were uploaded and published in the portal of MDM, i.e., are freely available.

The tool “CDEGenerator”, was used to compare semantic annotation and identify a semantic core via frequency analysis [32]. With the tool “ODMSummary”, the amount of ItemGroups was determined [33]. A medical expert manually reviewed the results and generated a catalog of disease related, i.e. domain-specific data elements. Certain data elements which are primarily available as structured data in the EMR, such as gender, age, administrative data and general lab results, were discarded. According to the U.S. National Library of Medicine (NLM) definition of CDEs only the “domain-specific” elements were considered [12]. This approach narrowed the extensive material of the NINDS CDEs down to the MS-specific “Diagnosis and disease characteristics” MS CDEs as source material beforehand. Clinical neurologists of the University Hospital Münster discussed the data elements and finalized a set of CDEs with a focus on clinical implementation and feasibility in clinical routine. To evaluate secondary use of the data, an item intersection analysis, displaying the intersection between the developed NID CDEs and the source documentation was performed. The illustration was generated using the R-package VennDiagram [34].

It is important to note, that although the entire source material has been converted, annotated and was used in the frequency analysis, during the manual review process by clinical neurologists, some elements were considered to be more important than others. Thus, our domain experts adjusted and completed the final data elements. Especially, data elements regarding the magnetic resonance imaging (MRI) were prioritized, although their frequential appearance was rather low in the source material.

### Integration into clinical workflow and pilot testing

The NID CDEs were initially implemented in the EMR (ORBIS from Agfa HealthCare) of the University Hospital Münster. “ORBIS Composer” was used to create an form in the EMR [35]. Focusing on a clear layout, the implementation included dynamic presentation of conditional data elements based on previous answers. For teaching purposes, the display of definitions for score values was integrated. Additionally, several automatic features were introduced to reduce documentation effort. However, a feasible implementation requires the analysis of clinical documentation workflows. Analysis was performed by attending the admission procedure of in- and outpatients with NIDs as well as interviewing physicians about their workflow.

For pilot testing the form in clinical routine, allowing changes and improvements, a six-month test phase was initiated at the University Hospital Münster. Tests were performed by neurologists in an inpatient and outpatient setting as well as in a neurology study center setting. Patients’ medical histories with diagnoses of MS, NMOSD, ADEM or related syndromes were documented with the NID CDEs. Primary purpose of data collection was obtaining medical history. Secondary purpose was creating the diagnosis block of the discharge letter. Clinicians were advised to report any inconsistencies and ambiguous questions. Minor adaptions concerning arrangement and clearer definition of data elements as well as completeness of answer options were performed. Afterwards the NID CDEs were additionally implemented at the University Hospital Köln.

### User evaluation, documentation completeness and generalization

To evaluate the usability of the implementation of NID CDEs in the EMR, a two-phase survey, containing the System Usability Scale (SUS) by Brooke [36], three demographic questions and two questions regarding the subjective documentation time in minutes for in- and outpatients on the first and following visit, was performed. A German version of the SUS was used, which has been previously translated within a crowdsourcing project [37]. The survey was conducted using LimeSurvey [38]. Both surveys are published in the Portal of MDM [39, 40].

Via email, 46 neurologists from the University Hospital Münster and seven neurologists from the University Hospital Köln, involved in medical care of patients with NIDs, were invited to participate in the study. The first survey evaluated the “existing” system, meaning the documentation processes before the implementation of the CDEs. The second survey, evaluating the “new system” began two months later, when clinicians had the chance to practice and use the implemented NID CDEs.

To evaluate documentation completeness concerning the amount of patients with NIDs documented with the minimal core dataset, the database of the EMR was queried. The query was restricted to patients of the Neurology Department of the University Hospital Münster, who were seen between January 2017 and February 2018 and were coded with the ICD-10-GM diagnoses G35.* for “MS” and G36.* for “other acute disseminated demyelination”. The query targeted patients who met the aforementioned criteria and were either documented with the NID CDEs or who were missing the form to evaluate documentation completeness.

Since the source material for the NID CDEs development was mostly German, justified by the targeted German applications for secondary use, the portability of the CDEs into other international systems was exemplary evaluated on MSBase. MSBase is an international online registry for neurologists studying multiple sclerosis and other neuro-immunological diseases with over 400 Members in over 30 countries [41]. New results in the filed of NIDs are frequently published based on this registry [42, 43]. The iMed software (Merck Serono SA—Geneva) is used as standard to collect data for MSBase. Thus, like described in the development of the minimal core dataset section, all domain-specific items of iMed have been annotated and the overlap with the final NID CDEs was determined.

## Results

### The minimal core dataset (NID CDEs)

The five document sources were transformed into ODM, semantically annotated and published in the Portal of MDM [44–48]. The UMLS codes for medical concepts were assigned according to the coding principles published by Varghese and Dugas [49]. Since partially, the UMLS Metathesaurus was missing codes, pinpointing the exact medical concept, alternative codes were used as shown in Table 1 [50].

**Table 1:** Medical concepts requiring an alternative UMLS coding due to the lack of accurate codes.

Medical concept in NID CDEs	Alternative coding	Concept meaning
Neuromyelitis optica spectrum disorders (NMOSD)	C0027873	Neuromyelitis optica (NMO)
NMOSD, monophasic	C4087481	Neuromyelitis optica spectrum disorder attack
NMOSD, relapsing	C4087551	Neuromyelitis optica spectrum disorder relapse
Longitudinal extensive transverse myelitis (LETM)	C0026976	Myelitis, Transverse
anti-MOG antibodies (Myelin oligodendrocyte glycoprotein)	C0003241	Antibodies
	C3266851	Myelin-Oligodendrocyte Glycoprotein
ADEM, monophasic	C0014059	Encephalomyelitis, Acute Disseminated

In total, 864 distinct medical concepts covering 1958 items across all source documents were identified. 424 of these medical concepts needed more than one UMLS code to represent their full medical meaning as been seen in Table 2. It is worth noticing that some items were annotated with more than a single concept. This results in more distinct concepts than items for REGIMS, biobank and MS CDEs. The reason for this behavior are items covering multiple concepts like “‘Are you pregnant or breast feeding?”’. Here the concept of pregnancy (C0032961) and breast feeding (C0006147) would have been applied.

**Table 2:** Distribution of items and distinct concepts of the source material.

Source	Items	Distinct concepts	Multiple UMLS codes
Discharge letter (12)	1284	431	151
REGIMS	161	164	104
Biobank	40	43	21
MS CDEs	61	72	51
DIMAT & ALAIN	412	305	154
Total	1958	864 (N = 1015)	424 (N = 481)

**Fig 1:**
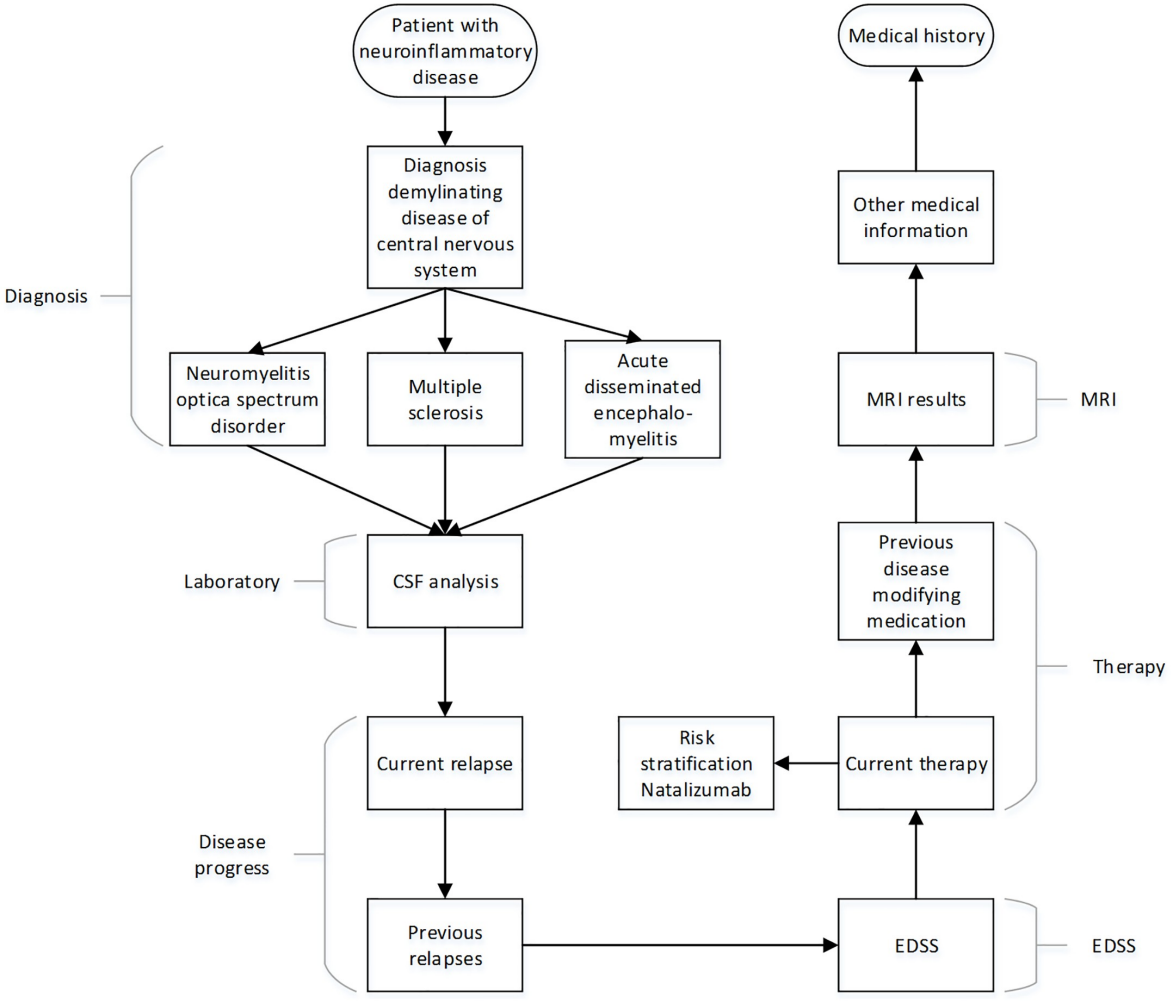
Schematic overview of the NID CDEs’s structure. 13 ItemGroups of the NID CDEs in chronological order for obtaining a patient’s medical history. Starting with documentation of diagnosis and confirmation of diagnoses (CSF analysis), followed by disease activity (disease progress, relapses and EDSS), current and previous disease modifying therapy and imaging results (MRI results). “Other medical information” provides the possibility of recording further medical information, such as secondary diagnoses, concomitant medications or findings in physical examination.

The 145 most frequent medical concepts covered 50% of all analyzed data elements. Seventy-five of these occurred in the discharge letters as well as in at least one other source. Discussion and review by participating clinicians and epidemiologists resulted in the NID CDEs with its ItemGroups displayed in Fig 1. With focus on clinical feasibility, 46 CDEs were selected, narrowing the amount of data elements to the most relevant ones occurring in clinical routine care. The data elements of NID CDEs can be categorized in three categories: “obligatory” (n = 11), “conditional but obligatory” (n = 35) and “optional” (n = 1). The category “conditional but obligatory” represents data elements that will not necessarily be relevant for every patient, depending on the constellation of diagnoses, therapies and preliminary exams.

Fig 1 shows order and conditionality of the ItemGroups of the NID CDEs. A detailed tabular view of ItemGroups, Items, CodeLists and occurrences in source documents is available as supplementary material (see S1 Table). The NID CDEs are published in English and German in the portal of MDMs and may be reused and translated for further academic purposes [51].

The NID CDEs contains six major sections, capturing information about diagnosis, laboratory results, disease progress, expanded disability status scale (EDSS) grading, therapy and MRI findings. Additionally, an item for further medical information, not covered by the NID CDEs, for instance, the documentation of concomitant diseases and therapies, has been developed. Overall, the NID CDEs consists of 13 ItemGroups, 46 Items with 23 CodeLists and 138 CodeListItems.

**Fig 2:**
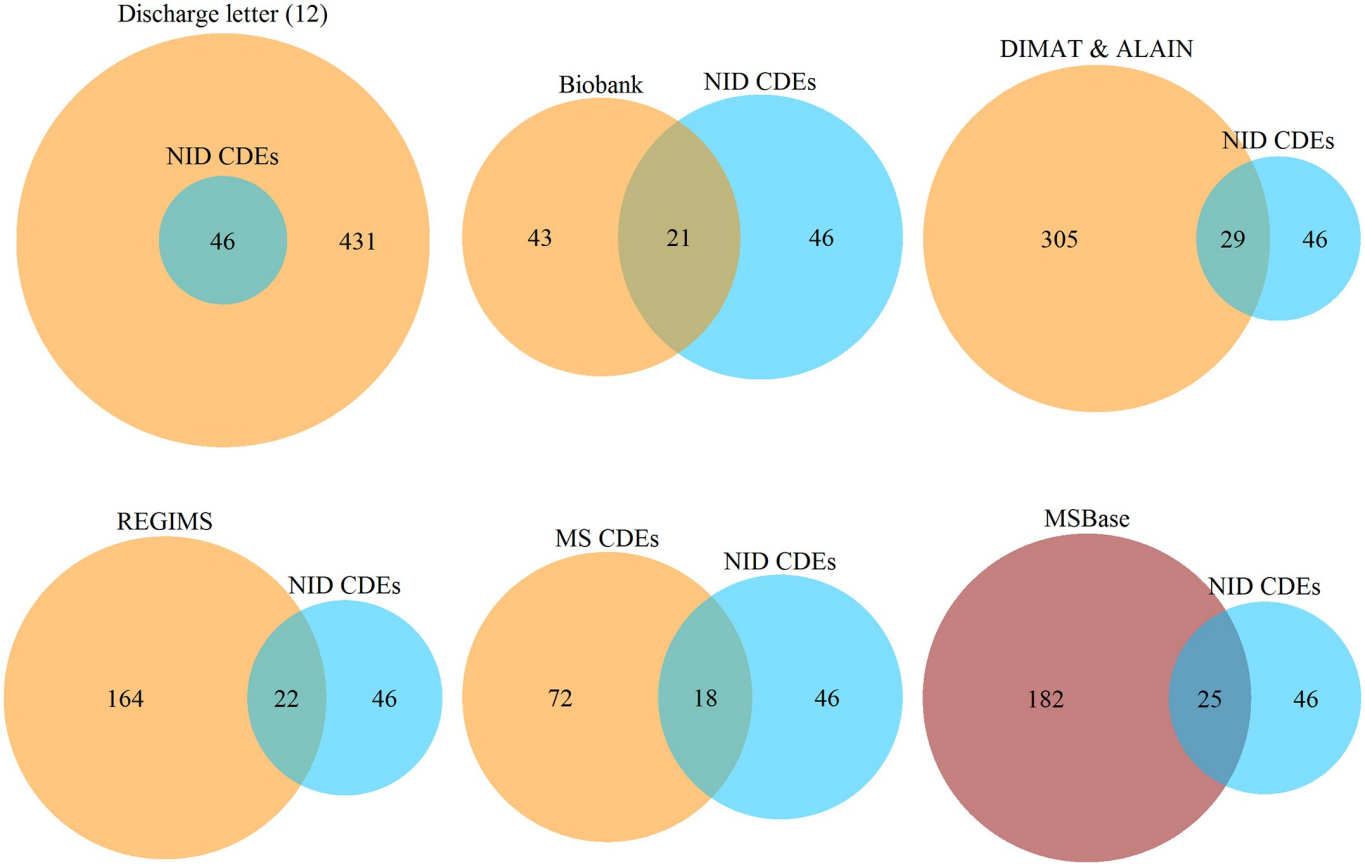
Two-set venn diagrams illustrating the intersection of distinct medical concepts with NID CDEs. All numbers indicate distinct medical concepts. The orange circles represent the source documentation forms, while the red one represents the MSBase registry, not been involved in the NID CDEs creation.

To identify the amount of items, which could be used for secondary purposes, an item intersection analysis was performed. Fig 2 shows the intersection of medical concepts as two-set Venn diagrams, comparing the source documentation forms (orange) with the developed NID CDEs (blue). The size of the circles represent the size of the dataset and the intersection of circles shows the amount of identical medical concepts. The red circle represents the MSBase documentation which was not considered during the NID CDEs development. For example all 46 data elements (DEs) of NID CDEs are to be found in the discharge letters that consist of a total of 431 distinct concepts. In comparison 21 concepts of the biobank are represented by the NID CDEs, the further 25 concepts are not to be used for the biobank. It is important to note that the distinct concepts of DCs and DIMAT&ALAIN represent an aggregation of the source material.

### Clinical workflow and integration of NID CDEs

**Fig 3:**
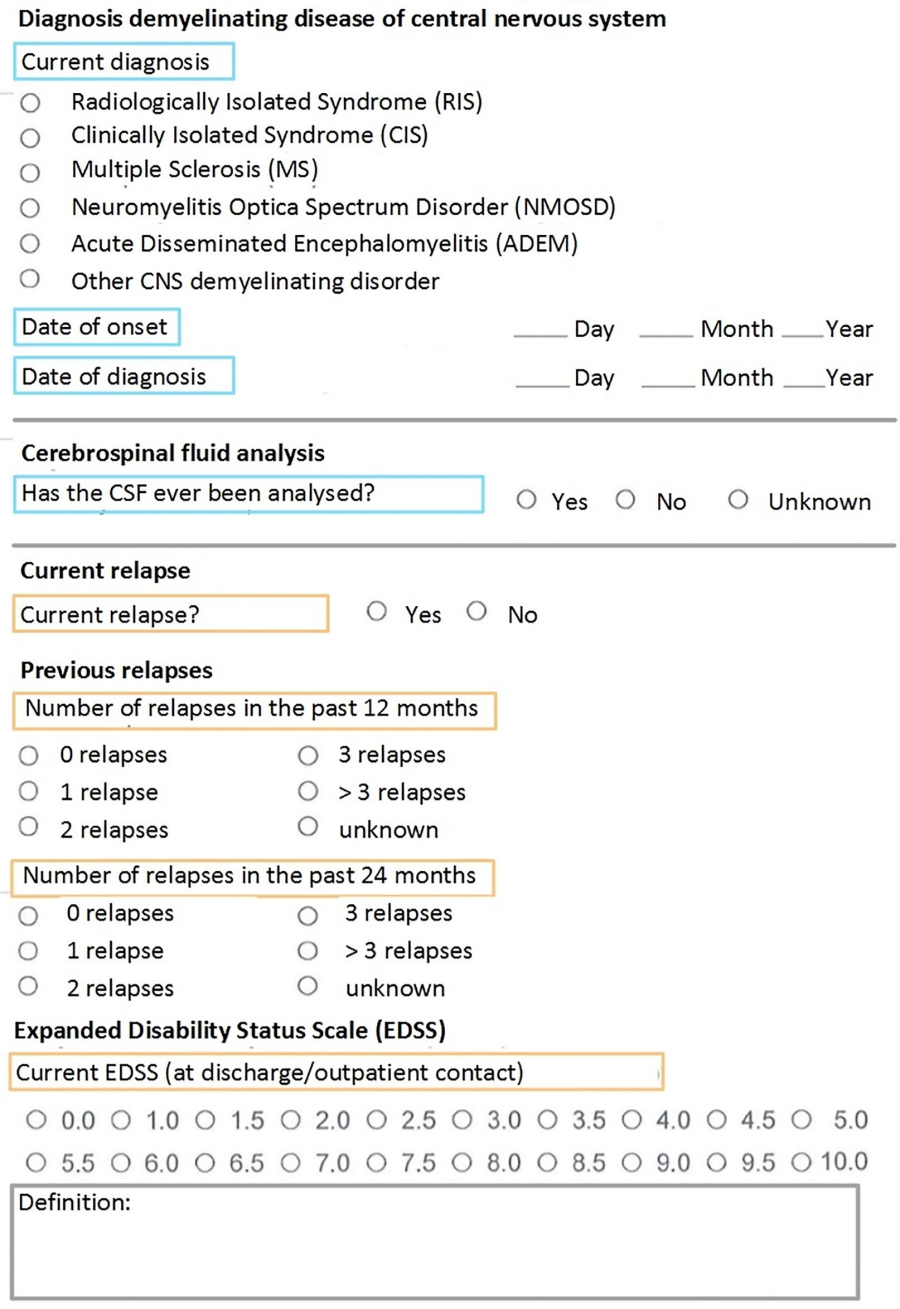
Screenshot of the minimal core dataset implemented as an EMR form. The Items were translated in English for illustration purposes. In follow-up patient contacts, the blue colored rectangles represent Items and ItemGroups, which are automatically prepopulated with data from the first contact. They only require editing if any changes happened in the meantime. Orange rectangles represent Items and ItemGroups that need to be collected in every patient contact.

The implemented form for structured documentation of the NID CDEs was integrated into the EMRs of the University Hospitals Münster and Köln. Fig 3 shows a screenshot of the implemented form. Analysis of the workflow showed, that patient contact with patients suffering from NIDs occurred in three different settings: The inpatient setting, leading to the admission of patients to the ward with inpatient treatment. The outpatient setting, allowing the follow-up and planning of further treatment options. Finally, the outpatient scheduling of study patients to include patients in clinical trials and carry out follow-up exams.

The clinical workflow before implementation of NID CDEs was performed as follows: After admission, inpatients and outpatients were seen by their treating physician. Medical history and physical examination were documented paper-based and transferred to the EMR. Discharge letters were either directly typed or dictated by the treating physician and forwarded to the writing service. The completed discharge letter then was revised, saved in the EMR and sent to the patient.

Possibilities for secondary use of data was limited to manual extraction of information. The biobank manager manually screened patients’ discharge letters and entered data into the documentation system of the neuroinflammatory biobank. To include patients into the multiple sclerosis registry, i.e., REGIMS, physicians captured the diagnosis-specific medical history in a separate paper-based form and forwarded it to study nurses to populate the data into the electronic data capture system. To identify patient cohorts meeting eligibility criteria of clinical trials, clinicians approached medical computer scientists to extract data from the EMR. Often, relevant data was only found in the free text section of discharge letters, impeding the accurate identification of patients and extraction of data.

**Fig 4:**
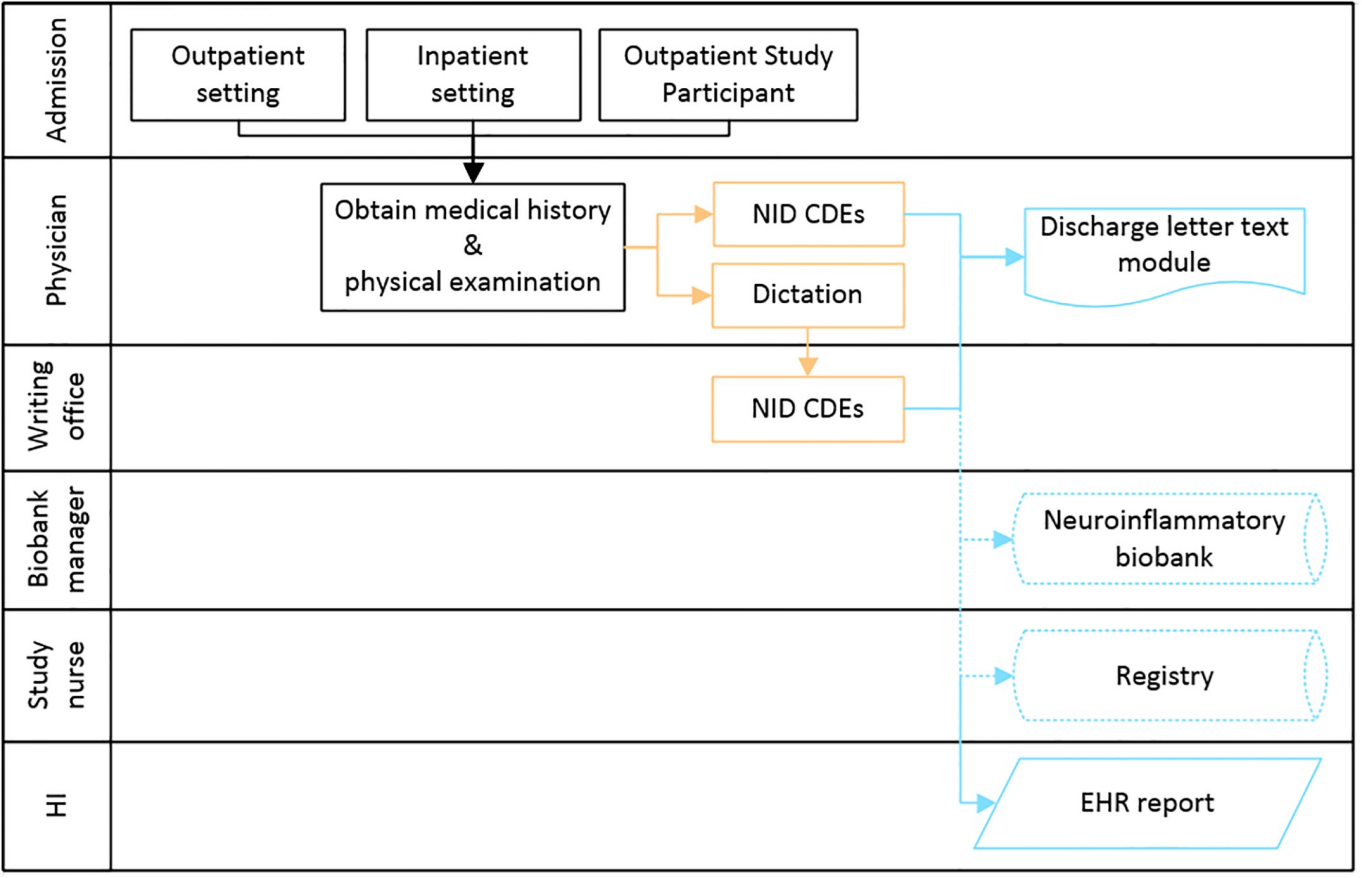
Clinical workflow after implementation of NID CDEs. Showing functional areas (Admission center, physicians, typing office, biobank manager, study nurse and health informatics (HI)) and the corresponding work steps. The black arrows and boxes represent manual steps, which have not been altered by the implementation of the NID CDEs. The orange arrows and boxes represent manual steps; the blue arrows represent automated steps. Dotted lines represent future steps that were considered during implementation, but still need to be implemented.

The previously described workflow changed after implementation of NID CDEs. Fig 4 illustrates the new workflow performed when seeing patients. Physicians now enter findings directly into the form for NID CDEs. If physicians dictate findings, the writing office enters dictated data into the form. Upon saving the form, a text module containing the collected data is automatically generated. The text module containing the diagnosis block may be retrieved and integrated into the discharge letter. Adhering to the original structure of the discharge letter, the text module contains current diagnosis and diagnosis-specific information. By storing structured information, medical computer scientists can easily query data that is relevant to identify patient cohorts or data for retrospective studies. The semi-automatic population of the structured information to the biobank and registry is not yet implemented as indicated by the dotted lines in Fig 4.

Several form functions were implemented to support fast, complete and consistent data collection. Functions are available depending on the documentation status. If a form is populated for the first time for a patient, the following functions are available:

- Display of EDSS value definitions for teaching purposes.
- Generation of the diagnosis block of the discharge letter.
- Sorting previous and current medications by ascending application date.

A form opened to document the follow-up during the next patient contact, offers the following additional functions:

- Prepopulated fields from previous contact (see Fig 3).
- Documentation of discontinuation of treatment with one click, shifting the drug from the “current treatment” section to the “previous treatment” section.
- Display of previous EDSS value and previous relapse frequency with their date. These values are not prepopulated because they might have changed since the last visit.

### Clinical usability and documentation completeness

In total, 19 neurologists participated in the user evaluation, assessing the clinical workflow before the implementation of NID CDEs and 19 neurologists assessed the clinical workflow after the implementation; 16 of them participated in both evaluations. Of these, 15 filled in the demographic questions (female n = 9, male n = 5, one without answer). With six senior neurologists, two neurology specialists and seven junior doctors, feedback was received from all user groups. The previously existing workflow was evaluated by 13 users, who rated the system with an average SUS score of 57, varying between 35 and 75 (Median 55). For the second part of the survey evaluating the new workflow, 16 users provided a mean SUS score of 75, varying from 50 to 97.5 (Median 75). Eleven neurologists participated in both parts of the survey. Only two of them rated the new workflow worse than the previously existing one.

According to Bangor et al. (2008), a system reaching a score greater than 72.75 is to be considered as a “good” usability score, whereas systems with a score greater than 52.01 are to be considered as “OK” [52]. Hence, the workflow was improved by implementing the NID CDEs from a “low marginal acceptable” workflow to an acceptable, good usable workflow.

Regarding the subjective estimation of documentation times, only eight of the 16 participants answered these questions. Two participants answered the question as range, i.e., interval, which was a design flaw in our survey to allow free text answers, and were ignored for the further calculations. Since not all six remaining participants had contact with in- and outpatients, the final answer count is reduced to four for outpatients and five for inpatients. An overview of all estimated times can be seen in Table 3. Except for the estimated time at the first visit of outpatients, all average documentation times decreased. However, the minimal and maximal estimation increased in all scenarios of the implemented NID CDEs.

**Table 3:** Subjectively estimated documentation times before and after the implementation of NID CDEs. Depicted times in minutes: Average (Minimum–Maximum).

	Outpatient (N = 4)		Inpatient (N = 5)	
	first-time	recurring	first-time	recurring
**Before**	12.50 (5–20)	6.75 (2–10)	18.0 (10–30)	8.0 (5–10)
**After**	18.75 (10–40)	6.00 (2–15)	17.4 (7–40)	5.8 (2–15)

Up to February 2018, 1725 forms for 755 patients with the ICD-10-GM diagnosis codes G35 and G36 were created. 1635 forms documented MS, followed by 50 NMOSD and 27 clinically isolated syndrome (CIS). The lowest amount of forms was collected for patients with radiologically isolated syndrome (RIS) and ADEM (RIS n = 1, ADEM n = 1). “Other CNS demyelinating disorder” was documented for 10 patients.

**Fig 5:**
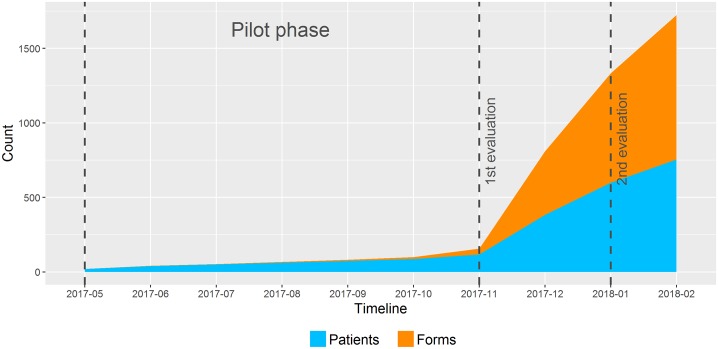
Cumulative number of patients and forms documented with the minimal core dataset. Numbers were retrieved from May 2017 to February 2018 in the University Hospital Münster. From May 2017 to November 2017, the form was pilot tested. In November 2017 the first evaluation of the previously existing system was performed and the form was used in clinical routine. The second evaluation of the new system was performed in January.

Fig 5 shows the cumulative number of forms and patients with NIDs documented with the minimal core dataset within nine months. In November 2017, a medical student was hired to retrospectively fill in the NID CDEs for patients back to January 2017 in order to support reusability of data and reduce documentation effort in follow-up visits. The difference between the number of patients and the amount of forms is due to the follow-up visits during this period, leading to multiple forms for one patient. Documentation completeness analysis showed that 755 of 905 (83,4%) patients and 1725 of 2139 (80,6%) of the cases (repeated visits of a patient) with the diagnoses G35.* and G36.* were documented with the NID CDEs.

## Discussion

### Related work

The NID CDEs were generated based upon source material from routine documentation but also CRFs and internationally accepted data standards, analyzing the semantic core of multiple sources. This method of generation of CDEs was applied before [2, 32, 53], but the developed CDEs have not been implemented for use in clinical routine. The generation of CDEs requires interdisciplinary collaboration and the cumbersome work of bringing experts together on one table and reaching an agreement on the most important data elements. Most standardization initiatives depend on the honorary participation of researchers. By using this technical approach, initiatives may save time and costs by proposing a semi-automatically generated set of data elements to experts, who will not have to ponder about every single item from scratch, but may rely on the core of previously used items. All required tools can be accessed online [28, 33, 54].

A huge amount of medical concepts was identified during analysis of the source documentation. Out of a list of 864 distinct medical concepts, only 46 were selected to be included into the NID CDEs. During integration of new documentation workflows in clinical routine, documentation time and the clinicians’ workload play a crucial role. Hence, the CDEs were developed with a focus on clinical practicability leading to a short list of the most important data elements for clinical routine, which can be reused for various research questions in current and future projects.

The NINDS developed a first set of CDEs for MS in 2011 and recommended using them for all MS studies. In addition to our local documentation forms, only the MS-specific “Diagnosis and disease characteristics” CDEs were considered during analysis and identification of the semantic core. This only represents a small excerpt of the very detailed study CDEs, which explains the low intersection of 18 data elements between our developed NID CDEs and this part of the source documentation. According to the diagnosis and disease characteristics for multiple sclerosis common data elements from the National Institute of Neurological Disorders and Stroke (MS CDEs) catalog, more than 1600 distinct CDEs have been developed to document MS in clinical trials funded by NINDS, of which about 20 items are declared as core or highly recommended, depending on the study type [55]. These 20 items are not necessarily disease-specific, but also contain core items such as birth date, ethnicity and gender [8]. With our focus on developing a minimal disease-specific core dataset for routine documentation, these core items were not applicable. As they were developed for the purpose of documentation in clinical trials, they could not be reused for routine data.

The six main parts identified, i.e., compare Fig 1, resemble the five dimensions for a patient-oriented five-dimensional approach for surveillance and therapy (FAST) in multiple sclerosis, published by Yalachkov et al. in 2017 [56]. Interestingly, almost every item mentioned in FAST is represented in the NID CDEs, except for the McDonald criteria [57, 58]. The McDonald criteria were not mentioned in the discharge letters and did not play a role in the registry or the neuroinflammatory biobank. Only the CRFs requested documentation of the McDonald criteria, confirming the diagnosis multiple sclerosis. Consulting neurologists, we decided to omit the McDonald criteria, focusing on the implementation of a small core amount of CDEs for clinical routine, and not serving the full implementation of items for CRFs of clinical trials.

### Feasibility and secondary use

This study shows the feasibility of generating CDEs by comparing different source materials and implementing the CDEs as a form into the clinical routine workflow. We defined CDEs to collect harmonized, structured data from patients with NIDs, reusing it for the diagnosis block of the discharge letter and maintaining it for further purposes such as patient cohort identification, transfer to a registry as well as a neuroinflammatory biobank.

By implementing the NID CDEs in the EMRs on two different sites, feasibility of integration into the clinical workflow, even in a multicentric setting, was evaluated after development. Following feedback of users during testing phase, the value domains were expanded and minor bugs eradicated. The user evaluation showed a good usability of the integrated NID CDEs, compared to the usability of the workflow before, rated as “ok”. Two clinicians rated the previous workflow better than the new one. A point of criticism was that collecting data in a structured way requires more time then taking notes by hand. Especially, at the first visit for patients who have a long disease history with numerous previous therapy regimes. This is reflected in Table 3, where the maximal time estimated for the first visit documentation is increased up to 40 minutes. However, in the following patient contacts, the collected data may be reused, leading to a reduced documentation effort as the average estimated times may indicate. In addition, since November 2017 a medical student creates the forms for old patient visits and enters existing data from previous discharge letters to facilitate documentation of patients with regularly scheduled appointments. Thus, the previous criticism of time consuming first-time documentation is omitted for recurring patients.

We have to point out, that unstructured notes may still be faster for recurring patients as the maximal estimated time in Table 3 shows. The structured data collection forces the physician to document items, which may not be important for the current clinical treatment and are therefore not collected for routine medical care. Ensuring the presence of certain structured information for all patients with diagnosis G35.* and G36.* enables scientific reports with very low percentages of missing data of information buried in discharge letters requiring natural language processing. In particular, since the data elements and source material were selected with focus on secondary use cases as depicted in Fig 4. Therefore, it is not surprising that the NID CDEs show an overlap with all targeted export systems (compare Fig 2). It is worth mentioning that the real number of exportable items will the higher than the figure may suggest. Universal data elements, like birth date or gender, although not covered by the CDEs, are present in the EMR and can be exported as well. Especially for discharge letters, the entire NID CDEs can be used secondary in the diagnosis block.

However, the focus on German source material with German secondary use cases rises the question of generalization and application of the CDEs to other international systems. Therefore, the disease-specific overlap with MSBase was determined as illustrated in Fig 2. From 182 distinct concepts stored in MSBase, NID CDEs covers at least 25. Thus, the minimal core dataset also facilitates the secondary use to international systems not considered during the development phase.

Furthermore, storing structured data enables data base queries and the extraction of data from electronic medical records to support patient cohort identification in clinical trial recruitment.

### Limitations

The NID CDEs are published in the portal of MDMs as semantically annotated ODM files. Even though the internationally accepted standard for concept annotation is represented by the Systematized Nomenclature of Human and Veterinary Medicine (SNOMED), the coding was performed with UMLS, since SNOMED has not been licensed in Germany yet. However, UMLS contains a mapping from UMLS concept codes to SNOMED, allowing retrospective data integration.

During the annotation process, an appropriate code from the UMLS Metathesaurus was found for most medical concepts. Interestingly though, at the time of analysis, the Metathesaurus did not contain a code for RIS. In the meantime, with the newer version of the Metathesaurus in November 2017, the code for RIS was added to the database (C4324721), showing contemporary research interest in this field. A few medical concepts required coding by alternative codes, as existing codes could not pinpoint the exact meaning of the concept (compare Table 1). For instance, this is the case for NMOSD, which includes limited or partial forms of NMO, but also manifestations with associated systemic autoimmune diseases [59]. Another example is the generation of a code for “anti-MOG antibody”, which currently requires two codes to represent the full medical meaning. Other NID-specific medical concepts that were missing appropriate coding are longitudinal extensive transverse myelitis (LETM), and disease courses of ADEM and NMOSD. These should be included into UMLS in order to insure an unambiguous coding of these concepts, which will be important for future data integration and interoperability.

Rather small numbers and missing open-metadata policies hampered the inclusion of registries, or CRFs for patients with NMO and ADEM. This led to an overrepresentation of MS-specific documentation, and the manual research and inclusion of NMO- and ADEM-specific data elements. Also the previously mentioned focus on German source material may have an influence on the overall representation. Additionally, the frequency analysis did not highlight all relevant medical concepts. Several items were explicitly requested by clinicians, such as the Natalizumab risk stratification, 12-month- and 24-months-frequency of relapses, and structured documentation of anti-MOG antibodies.

### Future work

Future work includes linking the NID CDEs to local neuroinflammatory biobanks, and to pharmacovigilance registries such as REGIMS, in order to automatically transfer collected data. Additionally, the development of CDEs for other disease entities, based on the methodology described here, is planned. The methodology should be further evaluated to assess its potential for a standardized procedure to develop CDEs in general.

The NID CDEs are open to the public and may be reused for non-commercial purposes. While ODM may be directly imported into various research systems, the Portal of MDM supports interoperability and reusability via converting ODM files into different technical formats, such as REDCap [60] or the OpenClinica [61] import format. However, several functions, such as generating a text module, display of conditional items as well as automatically prepopulating certain data fields may not be integrated into ODM. These features depend on the EMR and need to be implemented locally. This unpleasantly reminds us of missing standardization in implementation methods, hampering the free exchange of documentation forms between hospitals using different EMRs. Even the exchange of self-developed forms between installations of EMRs from the same provider at different locations needs to be approved and unlocked by the suppliers.

Overall, the minimal core datasets collected should be studied with regards to the benefit in clinical routine care, and systematic data analysis of patients with rare diseases, such as NMOSD, ADEM and MS. As requested by Peeters in 2017, the NID CDEs could serve as the first line of a standardized minimal data collection to be reused in a standard format for metadata in clinical trials.

## Conclusion

This work presented a feasibility study of developing a minimal core dataset for structured routine documentation, supporting secondary use in multiple clinical research settings. The integration into the clinical routine at two German university hospitals was evaluated and showed a good usability of the implemented CDEs. The secondary use had been established by generating the diagnosis block of discharge letters in a structured way and to enable automated reports for further research questions. At the time of writing, the semi-automatic population of the standardized elements to a biobank and a registry has not been implemented yet, but will be accomplished in near future.

The developed NID CDEs were published in a standard format in medical research, supporting sharing, translating, and reusing the CDEs. This can be the starting point for transnational harmonized documentation of patients with NIDs supporting secondary use.

## Supporting information

S1 Table Composition of the implemented NID CDEs. A detailed tabular view (Excel) of the contained ItemGroups, Items, and CodeLists in the implemented NID CDEs. Also, for each Item its occurrences in the source material is highlighted.
(XLSX)

## Abbreviations

4C plan: collect, connect, complete, construct
ADEM: acute disseminated encephalomyelitis
CDASH: Clinical Data Acquisition Standards Harmonization
CDE: common data element
CDISC: Clinical Data Interchange Standards Consortium
CIS: clinically isolated syndrome
CRF: case report form
CSF: cerebrospinal fluid
DC: discharge letter
DE: data element
DMT: disease modifying therapy
EDSS: expanded disability status scale
EMR: electronic medical record
FAIR: findable, accessible, interoperable and reusable
FAST: five-dimensional approach for surveillance and therapy
ICD-10-GM: International Statistical Classification of Diseases and Related Health Problems, 10th Revision, German Modification
KKNMS: German Competence Network for Multiple Sclerosis
LETM: longitudinal extensive transverse myelitis
MDM: medical data model
MOG: myelin oligodendrocyte glycoprotein
MRI: magnetic resonance imaging
MS: multiple sclerosis
MS CDEs: diagnosis and disease characteristics for multiple sclerosis common data elements from the National Institute of Neurological Disorders and Stroke
NID: neuroinflammatory demyelinating disease
NID CDEs: common data elements for neuroinflammatory demyelinating diseases
NIH: National Institute of Health
NINDS: National Institute of Neurological Disorders and Stroke
NLM: U.S. National Library of Medicine
NMO: neuromyelitis optica
NMOSD: neuromyelitis optica spectrum disorders
ODM: Operational Data Model
RIS: radiologically isolated syndrome
SNOMED: Systematized Nomenclature of Human and Veterinary Medicine
SUS: System Usability Scale
UMLS: Unified Medical Language System
